# Comprehensive analysis of PHGDH for predicting prognosis and immunotherapy response in patients with endometrial carcinoma

**DOI:** 10.1186/s12920-023-01463-5

**Published:** 2023-02-20

**Authors:** He Zhang, Weimin Kong, Xiaoling Zhao, Yunkai Xie, Dan Luo, Shuning Chen

**Affiliations:** Department of Gynecological Oncology, Beijing Obstetrics and Gynecology Hospital, Beijing Maternal and Child Health Care Hospital, Capital Medical University, 100006 Beijing, China

**Keywords:** PHGDH, Endometrial carcinoma, TCGA, Bioinformatics, Immune infiltrates

## Abstract

**Background:**

PHGDH (Phosphoglycerate Dehydrogenase) is the first branch enzyme in the serine biosynthetic pathway and plays a vital role in several cancers. However, little is known about the clinical significance of PHGDH in endometrial cancer.

**Methods:**

Clinicopathological data of endometrial cancer were downloaded from the Cancer Genome Atlas database (TCGA). First, the expression of PHGDH in pan-cancer was investigated, as well as the expression and prognostic value of PHGDH in endometrial cancer. The effect of PHGDH expression on the prognosis of endometrial cancer was analyzed by Kaplan-Meier plotter and Cox regression. The relationship between PHGDH expression and clinical characteristics of endometrial cancer was investigated by logistic regression. Receiver operating characteristic (ROC) curves and nomograms were developed. Possible cellular mechanisms were explored using the Kyoto Encyclopedia of Genes and Genomes (KEGG) pathway enrichment analysis, the Gene Ontology (GO), and gene set enrichment analysis (GSEA). Finally, TIMER and CIBERSORT were used to analyze the relationship between PHGDH expression and immune infiltration. CellMiner™ was used to analyze the drug sensitivity of PHGDH.

**Results:**

The results showed that PHGDH expression was significantly higher in endometrial cancer tissues than in normal tissues at mRNA and protein levels. Kaplan-Meier survival curves showed that patients in the high expression group had shorter overall survival (OS) and disease free survival (DFS) than patients in the low PHGDH expression group. Multifactorial COX regression analysis further supported that high PHGDH expression was an independent risk factor associated with prognosis in patients with endometrial cancer. The results showed estrogen response, mTOR, K-RAS, and epithelial mesenchymal transition (EMT) were differentially elevated in the high-expression group of the PHGDH group. CIBERSORT analysis showed that PHGDH expression is related to the infiltration of multiple immune cells. When PHGDH is highly expressed, the number of CD8^+^T cells decreases.

**Conclusion:**

PHGDH plays a vital role in the development of endometrial cancer, which is related to tumor immune infiltration, and can be used as an independent diagnostic and prognostic marker for endometrial cancer.

**Supplementary Information:**

The online version contains supplementary material available at 10.1186/s12920-023-01463-5.

## Background

Endometrial cancer is one of the three significant malignancies of the female reproductive system, with the highest incidence. More than 400,000 new cases of endometrial cancer were diagnosed worldwide in 2020 [1], and nearly 70,000 new cases will be diagnosed in the United States in 2021 [2]. It comprises a group of malignant epithelial tumors originating from the endometrium, 80% of which are endometrioid carcinomas, and is common in perimenopausal and postmenopausal women. In recent years, the incidence of endometrial cancer has gradually increased as economic conditions have improved and average life expectancy has increased. Data from the Cancer Genome Atlas (TCGA) project have deepened our understanding of the biological heterogeneity of endometrial cancer [3, 4]. However, challenges remain, and although survival rates for patients with endometrial cancer appear promising, there is still a need for clear and valid biological markers to aid in definitive diagnosis.

PHGDH (Phosphoglycerate Dehydrogenase) is a protein-coding gene [5, 6]. The enzyme encoded by this gene is involved in the early steps of L-serine synthesis in animal cells and is highly expressed in some tumors. Tumor cells are like a sizeable biosynthetic factory configured with multiple metabolic pathways associated with cell growth and reproduction. Serine is both protein-derived amino acid and a source of one-carbon units necessary for the de novo synthesis of purines and deoxythymidine [7]. Serine is also the third most relevant substance for cancer cell metabolism, after glucose and glutamate. Studies in the late 1980s demonstrated increased serine synthesis in cancer cells, implying that this pathway was associated with tumor growth [8].

Still, it was not until the later discovery of increased expression of the PHGDH gene in breast cancer and melanoma that researchers brought the PHGDH pathway into the light [7, 9]. Several studies in recent years have shown that PHGDH inhibitors selectively block the growth of PHGDH-dependent cancer cells and that silencing the PHGDH gene significantly affects the development of PHGDH-dependent cancers, making the enzyme a new target for cancer therapy [10]. Study by Shen et al. demonstrates that PHGDH inhibits bladder cancer ferroptosis and promotes malignant tumor progression through upregulation of SLC7A11 [11].

A study by Rossi et al. identified a heterogeneous or low expression of PHGDH associated with a shorter metastasis-free survival in breast cancer patients [12]. The mechanism of this phenomenon may be related to the interaction of PHGDH protein with the glycolytic enzyme phosphofructokinase (PFK). The absence of this interaction is able to activate the hexosamine-sialic acid pathway, which provides a precursor for protein glycosylation. The occurrence of aberrant protein glycosylation enhances the migration and invasion of tumor cells [12]. Thus, the presence of PHDGH heterogeneity in primary tumors may be considered a marker of tumor aggressiveness. The above evidence suggests that PHGDH may be a gene associated with poor tumor prognosis and can regulate the biological behavior of malignant tumors by promoting tumor cell migration, invasion, and inhibiting tumor cell death in various ways. However, how PHGDH affects the pathogenesis and prognosis of endometrial cancer remains to be further investigated.

This study aimed to determine the prognostic value of PHGDH in endometrial cancer using comprehensive bioinformatics analysis. First, PHGDH mRNA expression was evaluated in pan-cancer, endometrial cancer, and benign tissues. Then, the correlation between PHGDH expression and prognosis of endometrial cancer patients was analyzed. We then determined the correlation between PHGDH expression and clinical features in patients with endometrial cancer and used enrichment analysis bases to identify biological pathways associated with PHGDH. We developed a nomogram prediction model and analyzed the correlation between PHGDH expression and immune infiltration. Finally, we screened for PHGDH-related susceptibility drugs. Our results suggest that PHGDH may be a promising biomarker for the diagnosis and prognosis of endometrial cancer.

## Methods

### The TCGA database

PHGDH gene expression in endometrial carcinoma and the corresponding clinical information data were downloaded from the TCGA database (https://tcga-data.nci.nih.gov/tcga/) [13]. UCEC (Endometrioid Cancer) data included the clinical stage, tumor grade, pathological subtypes, age, and other patients’ data. This study analyzed PHGDH RNA-seq data and its association with the OS of patients with endometrial carcinoma in the TCGA-UCEC dataset. Patients with endometrial carcinoma were divided into high and low expression groups based on the median mRNA expression values. Normalization was performed when comparing RNA-seq data between samples. Data were collected and analyzed using R3.6.3 software [14].

### UALCAN database

UALCAN is a comprehensive and interactive web resource that provides easy access to publicly available cancer OMICS data (The Cancer Genome Atlas (TCGA), MET500, and Clinical Proteomic Tumor Analysis Consortium (CPTAC) databases and allows users to identify biomarkers or perform in silico validation of potential genes of interest (http://ualcan.path.uab.edu/index.html). Here, the mRNA and protein expression of PHGDH in endometrial carcinoma was evaluated using CPTAC databases [15].

### Immunohistochemical staining in the HPA database

The Human Protein Atlas (HPA, http://www.proteinatlas.org/) online database was explored to validate the PHGDH protein expression in endometrial cancer by immunohistochemical staining CAB003681 antibody [16].

### Kaplan–Meier plotter database

The prognostic value of PHGDH in endometrial carcinoma was assessed according to overall survival (OS) and disease free survival (DFS) using Kaplan–Meier plotter. Sources for the databases include Gene Expression Omnibus (GEO), The European Genome-phenome Archive (EGA), and TCGA (https://kmplot.com/analysis/). The tool’s primary purpose is the meta-analysis-based discovery and validation of survival biomarkers [17].

### GO, KEGG pathway enrichment analysis, and GSEA for PHGDH

This study generated an ordered list of genes based on the correlation between all genes and PHGDH expression using GO, KEGG, and GSEA. Data were collected and analyzed using R 3.6.3 software [14]. The Gene Ontology (GO) knowledgebase is the world’s largest source of information on the functions of genes (http://geneontology.org/) [18–20]. KEGG is a collection of databases dealing with genomes, biological pathways, diseases, drugs, and chemical substances (www.kegg.jp/kegg/kegg1.html) [21–23]. Genes were differentially expressed based on an absolute fold change > 1.5 and P_adj_<0.05.

GSEA is a computational method that allows the determination of classes of genes or proteins overrepresented in a large set of genes or proteins and may have a statistically significant association with disease phenotypes [24]. The predefined gene set is from the MSigDB database (https://www.gsea-msigdb.org/gsea/msigdb/index.jsp). This study generated an ordered list of genes based on the correlation between all genes and PHGDH expression using GSEA. The enriched pathways were determined based on the nominal P-value and the normalized enrichment score (NES).

### TIMER analysis and the CIBERSORT package

Tumor immune to assess resource (TIMER) is a reliable and convenient database, including gene expression profiles from the TCGA database (https://cistrome.shinyapps.io/timer/) [25, 26]. TIMER tool can estimate immune cell infiltration and assess its clinical impact. The somatic copy number alteration (SCNA) module of the TIMER tool links the genetic copy number variation (CNV) of PHGDH to the relative abundance of tumor-infiltrating cells. The CIBERSORT package was used to assess the relative proportions of 24 immune infiltrating cells in tumor samples when PHGDH was highly or lowly expressed [27].

### Drug sensitivity of PHGDH

Our study downloaded drug sensitivity processing data from the CellMiner™ database (https://discover.nci.nih.gov/cellminer/home.do). It is a database and query tool designed for the cancer research community to facilitate the integration and study of molecular and pharmacological data for the NCI-60 cancerous cell lines [28, 29]. All data were processed using the R-packages “impute”, “limma” [30], “ggplot2” [31] and “ggpubr” [32] for analysis and visualization.

### Statistical analysis

The Pearson χ2 test was used to analyze the association between PHGDH expression and clinicopathological characteristics, and the FISHER exact test was used when necessary. OS was defined as the time from random assignment to death from any cause, and DFS was defined as the chances of staying free of a disease or cancer after a particular treatment. Survival analysis was performed using the Kaplan-Meier method, and statistical treatment was performed using the log-rank test. Using univariate Cox proportional hazards regression, individual hazard ratios (HR) for OS and DFS were estimated.

COX proportional hazards regression was used to analyze the independent prognostic factors affecting the prognosis of patients with endometrial cancer. Statistical analysis was performed using R3.6.3 software. A chi-square test was used to compare the clinicopathological profile of the high and low expression groups. The 95% confidence interval (CI) of HR was measured to estimate the risk of individual factors. Nomograms were plotted with R package “rms” [33] to create predictive models. P < 0.05 was considered statistically significant, and p < 0.01 was considered highly statistically significant. All reported P values are bilateral.

## Results

### The relative expression level of PHGDH in endometrial cancer

**Fig. 1:**
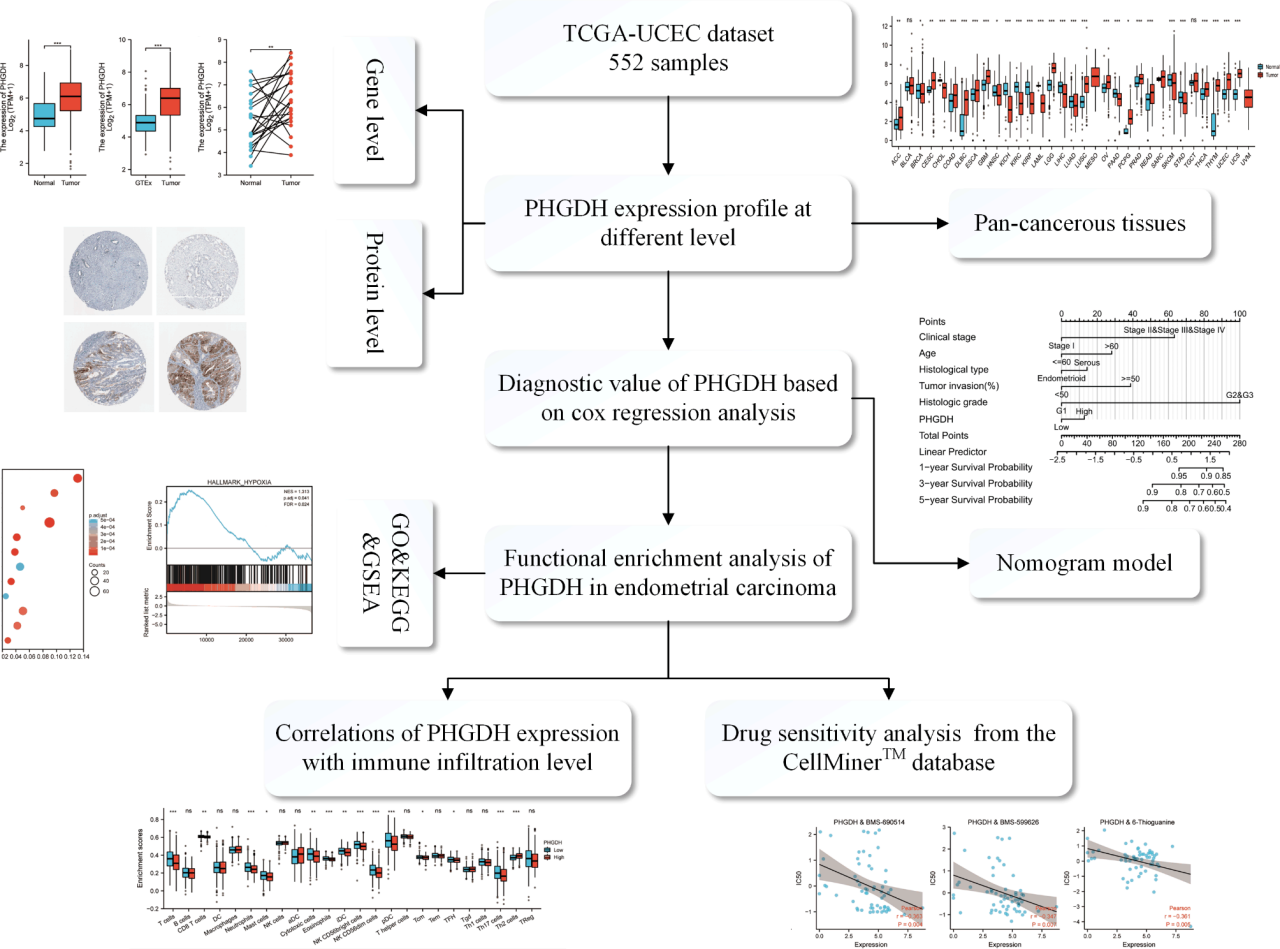
Flow chart of the relationship between high PHGDH expression and prognosis in EC

**Fig. 2:**
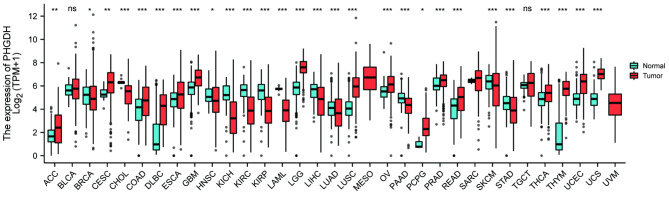
Expression levels of PHGDH genes in pan-cancerous tissues in the TCGA database

The flow chart of this study is shown in Fig. 1. First, we summarize the expression levels of PHGDH mRNA in pan-cancer (33 cancers) from the TCGA database (Fig. 2). Among them, the expression level of PHGDH in tumor tissues was significantly higher than that in normal tissues, including endometrial cancer tissues (P < 0.001).

**Fig. 3:**
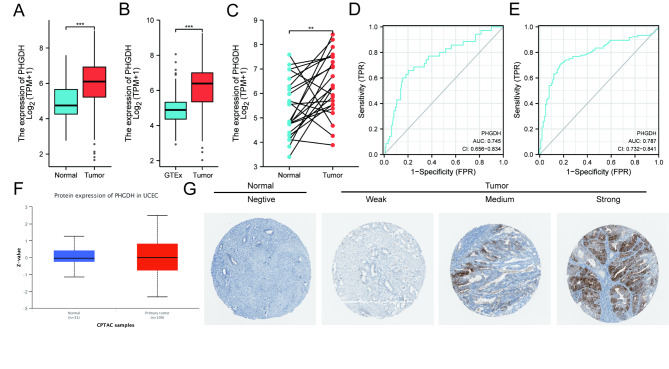
High PHGDH expression is correlated with clinicopathologic features in patients with endometrial carcinoma. (A) Differences in PHGDH expression in TCGA-UCEC tissues and adjacent normal tissues. (B) Differences in PHGDH expression in normal samples from the GTEx and TCGA-UCEC samples. (C) Differences in PHGDH expression in TCGA-UCEC samples and paired adjacent samples. (D, E) ROC curve for Fig. 3A,B. (F) PHGDH protein levels were markedly upregulated in tumor tissues compared with that in non-paired normal tissues. (G) Representative immunohistochemistry images of PHGDH expression in endometrial carcinoma tissues and their normal controls

Next, we explored the relationship between PHGDH expression and clinicopathological features of endometrial cancer. Next, to investigate the role of PHGDH in endometrial cancer progression, we explored the relationship between PHGDH expression and clinicopathological characteristics of endometrial cancer. The expression pattern of PHGDH gene in 552 cases of endometrial cancer tissues and 35 cases of normal endometrial tissues was predicted using the TCGA database (Fig. 3A). The results showed that the expression level of PHGDH gene in primary endometrial cancer tissues was significantly higher than that in normal endometrial tissues (P < 0.001). The expression of PHGDH in normal tissues adjacent to cancer and UCEC tissues were further compared. PHGDH was highly expressed in UCEC tissues compared with paraneoplastic tissues (P < 0.001) (Fig. 3B). In addition, PHGDH expression was significantly upregulated in 23 cases of endometrial cancer tissues compared with paired paraneoplastic tissues (P < 0.01) (Fig. 3C).

To evaluate the diagnostic value of PHGDH expression levels in normal GTEx/UCEC tissues and paraneoplastic/UCEC tissues, we also plotted the receiver operating characteristic (ROC) curves separately. The area under the curve (AUC value) of PHGDH expression levels in normal GTEx/UCEC tissues was 0.745 (CI = 0.656–0.834), and the area under the curve (AUC) value for the expression level in paraneoplastic/UCEC tissues was 0.787 (CI = 0.732–0.841), suggesting a high diagnostic potential (Fig. 3D, E).

The protein expression of PHGDH was analyzed using the UALCAN and HPA databases. The protein expression level of PHGDH was also significantly upregulated in endometrial cancer tissues compared to normal endometrial tissues (Fig. 3F, G), indicating that PHGDH protein and mRNA had similar expression profiles in different databases.

### Correlation between PHGDH expression and clinical features

**Fig. 4:**
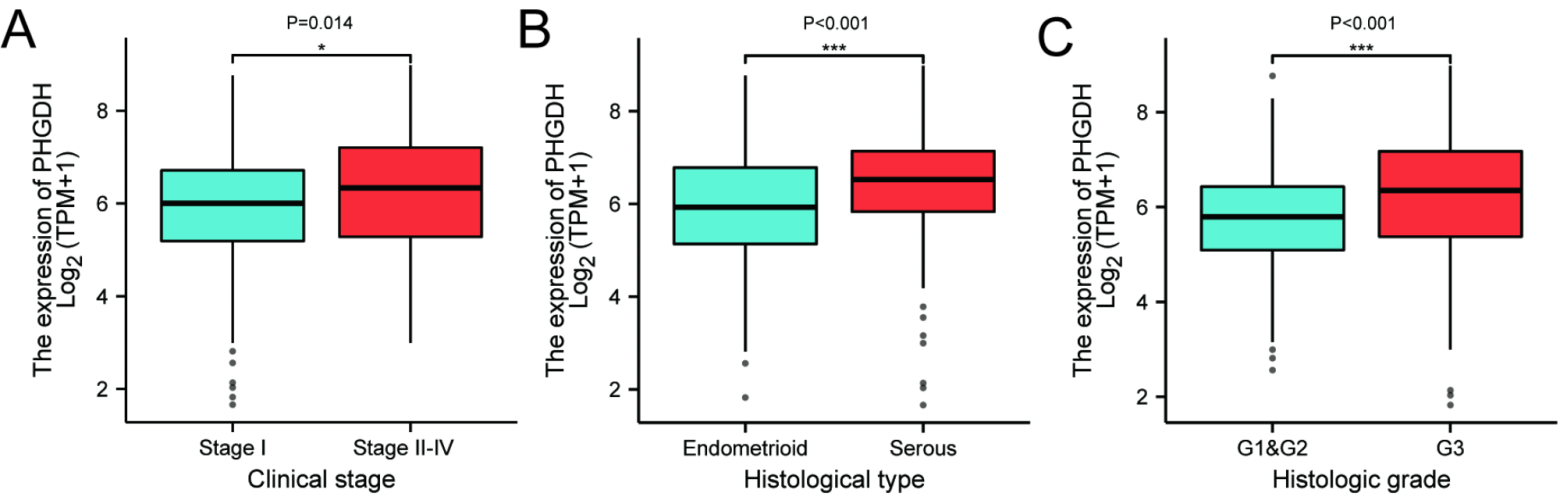
Box plot assessing PHGDH expression in patients with endometrial carcinoma according to different clinical characteristics. (A) Clinical Stage, (B) Histological type, (C) Histologic grade

We then analyzed the relationship between PHGDH expression and clinical characteristics in 552 patients with endometrial cancer in the TCGA-UCEC dataset (Table 1). Patients were divided into PHGDH high and low expression groups according to the mean value of PHGDH expression. The relationship between PHGDH expression and clinical characteristics was assessed using Wilcoxon signed rank test and logistic regression analysis. The results showed statistically significant differences in PHGDH expression between stage I and stage II-IV tumors (P = 0.014) (Fig. 4A) and between histological grade G1/2 and G3 (P < 0.001) (Fig. 4C). PHGDH expression was also higher in serous endometrial carcinoma than in endometrioid carcinoma (P < 0.001) (Fig. 4B).

**Table 1:** The relationship between PHGDH mRNA expression and clinical characteristics in endometrial carcinoma

Characteristic		Low expression of PHGDH	High expression of PHGDH	p
**n**		276	276	
**Clinical stage, n (%)**				0.085
	Stage I	183 (33.2%)	159 (28.8%)	
	Stage II	27 (4.9%)	24 (4.3%)	
	Stage III	55 (10%)	75 (13.6%)	
	Stage IV	11 (2%)	18 (3.3%)	
**Primary therapy outcome, n (%)**				0.006
	PD	7 (1.5%)	13 (2.7%)	
	SD	4 (0.8%)	2 (0.4%)	
	PR	1 (0.2%)	11 (2.3%)	
	CR	228 (47.5%)	214 (44.6%)	
**Race, n (%)**				0.905
	Asian	9 (1.8%)	11 (2.2%)	
	Black or African American	54 (10.7%)	54 (10.7%)	
	White	190 (37.5%)	189 (37.3%)	
**Age, n (%)**				0.238
	<=60	110 (20%)	96 (17.5%)	
	> 60	164 (29.9%)	179 (32.6%)	
**Weight, n (%)**				0.167
	<=80	114 (21.6%)	129 (24.4%)	
	> 80	152 (28.8%)	133 (25.2%)	
**BMI, n (%)**				0.578
	<=30	103 (19.8%)	109 (21%)	
	> 30	158 (30.4%)	149 (28.7%)	
**Histological type, n (%)**				< 0.001
	Endometrioid	227 (41.1%)	183 (33.2%)	
	Mixed	12 (2.2%)	12 (2.2%)	
	Serous	37 (6.7%)	81 (14.7%)	
**Residual tumor, n (%)**				0.994
	R0	184 (44.6%)	191 (46.2%)	
	R1	11 (2.7%)	11 (2.7%)	
	R2	8 (1.9%)	8 (1.9%)	
**Histologic grade, n (%)**				< 0.001
	G1	70 (12.9%)	28 (5.2%)	
	G2	69 (12.8%)	51 (9.4%)	
	G3	132 (24.4%)	191 (35.3%)	
**Tumor invasion(%), n (%)**				0.202
	< 50	138 (29.1%)	121 (25.5%)	
	>=50	101 (21.3%)	114 (24.1%)	
**Menopause status, n (%)**				0.688
	Pre	18 (3.6%)	17 (3.4%)	
	Peri	10 (2%)	7 (1.4%)	
	Post	221 (43.7%)	233 (46%)	
**Hormones therapy, n (%)**				0.786
	No	148 (43%)	149 (43.3%)	
	Yes	25 (7.3%)	22 (6.4%)	
**Diabetes, n (%)**				0.885
	No	161 (35.7%)	167 (37%)	
	Yes	62 (13.7%)	61 (13.5%)	
**Radiation therapy, n (%)**				0.825
	No	141 (26.8%)	138 (26.2%)	
	Yes	122 (23.1%)	126 (23.9%)	
**Age, meidan (IQR)**		63 (56, 71)	64 (58, 72)	0.183

Univariate logistic regression analysis showed the correlation between PHGDH expression and clinicopathological features of endometrial cancer (Table 2). A comparison of baseline information between the high and low PHGDH expression groups showed that PHGDH expression was associated with the clinical stage (Stage I vs. Stage II-IV, OR = 1.448, P = 0.036), primary therapy outcome (CR vs. PD&SD&PR, OR = 0.433, P = 0.021), histological type (Serous vs. Endometrioid, OR = 2.716, P < 0.001), and histological grade (G1&G2 vs. G3, OR = 2.546, P < 0.001) were significantly associated.

**Table 2:** Logistic regression demonstrates the correlation between PHGDH expression and clinicopathological features

Characteristics	Total(N)	Odds Ratio(OR)	P value
Clinical stage (Stage II-IV vs. Stage I)	552	1.448 (1.026–2.048)	0.036*
Primary therapy outcome (CR vs. PD&SD&PR)	480	0.433 (0.206–0.862)	0.021*
Age (> 60 vs. <=60)	549	1.251 (0.885–1.770)	0.205
Weight (> 80 vs. <=80)	528	0.773 (0.548–1.089)	0.142
BMI (> 30 vs. <=30)	519	0.891 (0.628–1.265)	0.519
Histological type (Serous vs. Endometrioid)	528	2.716 (1.770–4.232)	< 0.001*
Residual tumor (R1&R2 vs. R0)	413	0.963 (0.492–1.886)	0.913
Histologic grade (G2&G3 vs. G1)	541	3.010 (1.888–4.912)	< 0.001*
Tumor invasion(%) ( > = 50 vs. <50)	474	1.287 (0.896–1.851)	0.172
Menopause status (Peri&Post vs. Pre)	506	1.100 (0.551–2.203)	0.786
Diabetes (Yes vs. No)	451	0.949 (0.626–1.436)	0.803

### Correlation between PHGDH expression and clinical features

The independent diagnostic value of PHGDH expression in endometrial carcinoma.

**Fig. 5:**
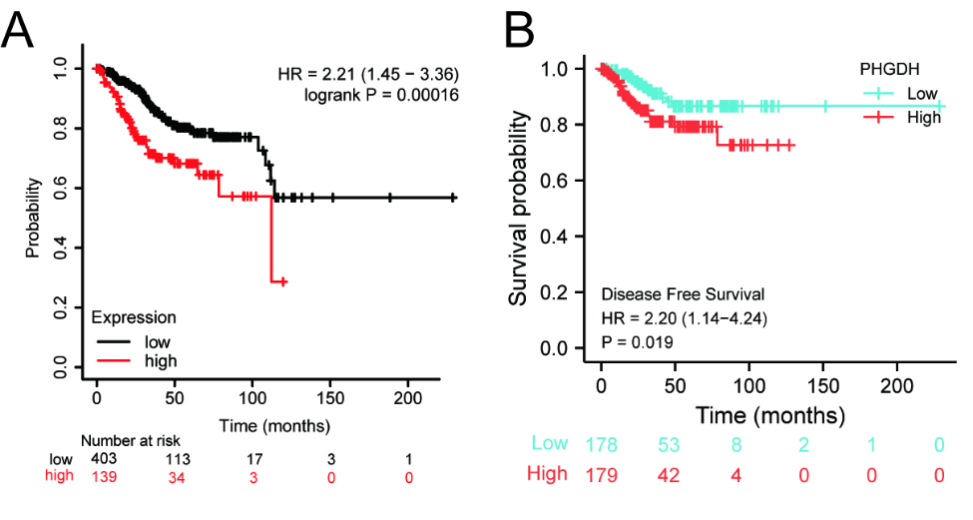
The independent risk and diagnostic value of PHGDH expression in endometrial carcinoma. (A) OS, (B) DFS

**Fig. 6:**
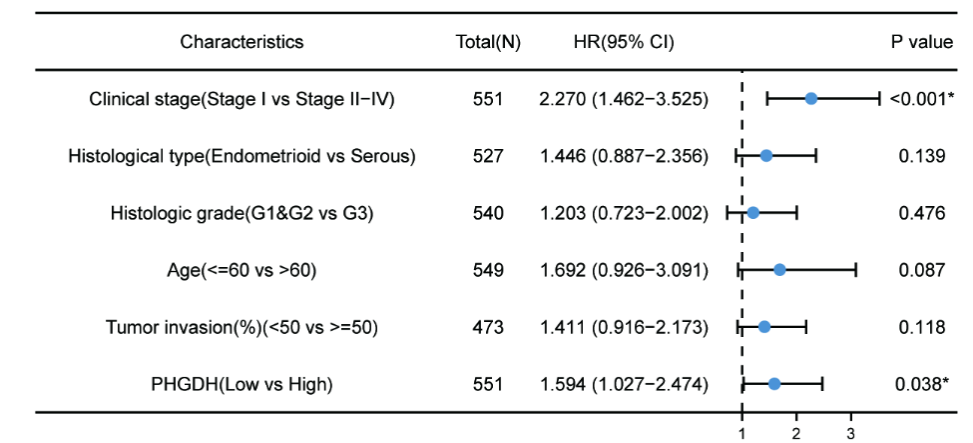
Forest plot of the multivariate Cox regression analysis in endometrial carcinoma patients

Survival analysis demonstrated that high PHGDH expression was correlated with poor OS (P < 0.001, HR = 2.21) as well as poor DFS (P = 0.019, HR = 2.20) (Fig. 5A, B). Univariate Cox regression analysis showed that high PHGDH expression was significantly correlated with poor OS (HR = 1.698, 95% CI = 1.112–2.592) and DFI (HR = 1.614, 95% CI = 1.132-2.300). Moreover, multivariate regression analysis and visualized forest plots further confirmed that PHGDH expression was an independent prognostic factor for DFI in patients with endometrial carcinoma (HR = 1.614, 95% CI = 1.027–2.474, P = 0.038) (Table 3; Fig. 6).

**Table 3:** Multivariate COX regression reveals the correlation between PHGDH and endometrial cancer survival

Characteristics	Total(N)	HR(95% CI) Univariate analysis	P value Univariate analysis	HR(95% CI) Multivariate analysis	P value Multivariate analysis
Clinical stage	551				
Stage I	341	Reference			
Stage III&Stage IV&Stage II	210	2.527 (1.780–3.587)	< 0.001	2.270 (1.462–3.525)	< 0.001
Histological type	527				
Endometrioid	409	Reference			
Serous	118	2.125 (1.466–3.081)	< 0.001	1.446 (0.887–2.356)	0.139
Histologic grade	540				
G1&G2	218	Reference			
G3	322	2.088 (1.391–3.136)	< 0.001	1.203 (0.723–2.002)	0.476
Age	549				
<=60	206	Reference			
> 60	343	1.353 (0.934–1.961)	0.110		
Tumor invasion(%)	473				
< 50	259	Reference			
>=50	214	1.885 (1.289–2.756)	0.001	1.411 (0.916–2.173)	0.118
PHGDH	551				
Low	275	Reference			
High	276	1.614 (1.132-2.300)	0.008	1.594 (1.027–2.474)	0.038
Menopause status	505				
Pre	35	Reference			
Peri&Post	470	1.530 (0.673–3.477)	0.310		

**Fig. 7:**
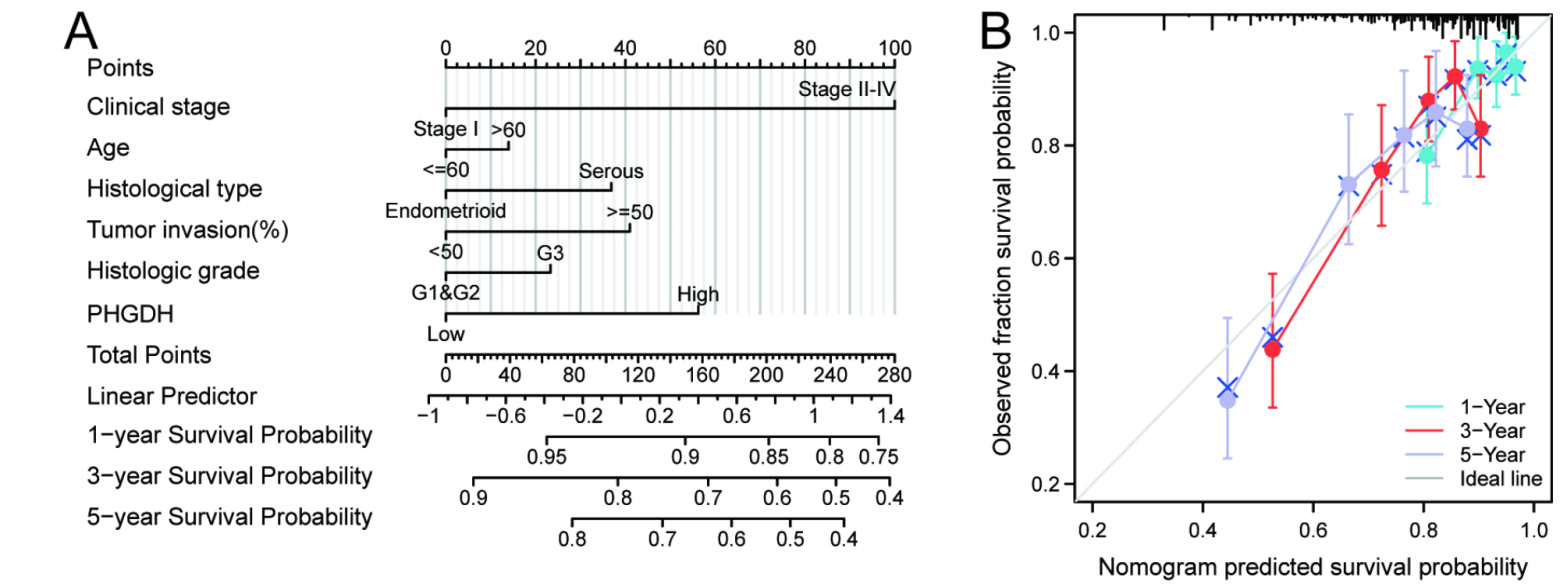
Construction and validation of nomogram based on PHGDH expression. (A) A nomogram for predicting the probability of 1-, 3- and 5-year OS in endometrial carcinoma patients. (B) Calibration plots validating the efficiency of nomograms for OS

Subsequently, nomogram models predicting the survival of patients with endometrial cancer were constructed using age, clinical stage, histological grade, tumor invasion, histological type, and PHGDH levels (Fig. 7A). The calibration curves provided ideal nomogram predictions for clinical outcomes at 1, 3, and 5 years (Fig. 7B). The above results suggest that PHGDH could be a valuable biomarker for predicting survival in patients with endometrial cancer.

### PHGDH expression-related signaling pathway based on GSEA and KEGG

**Fig. 8:**
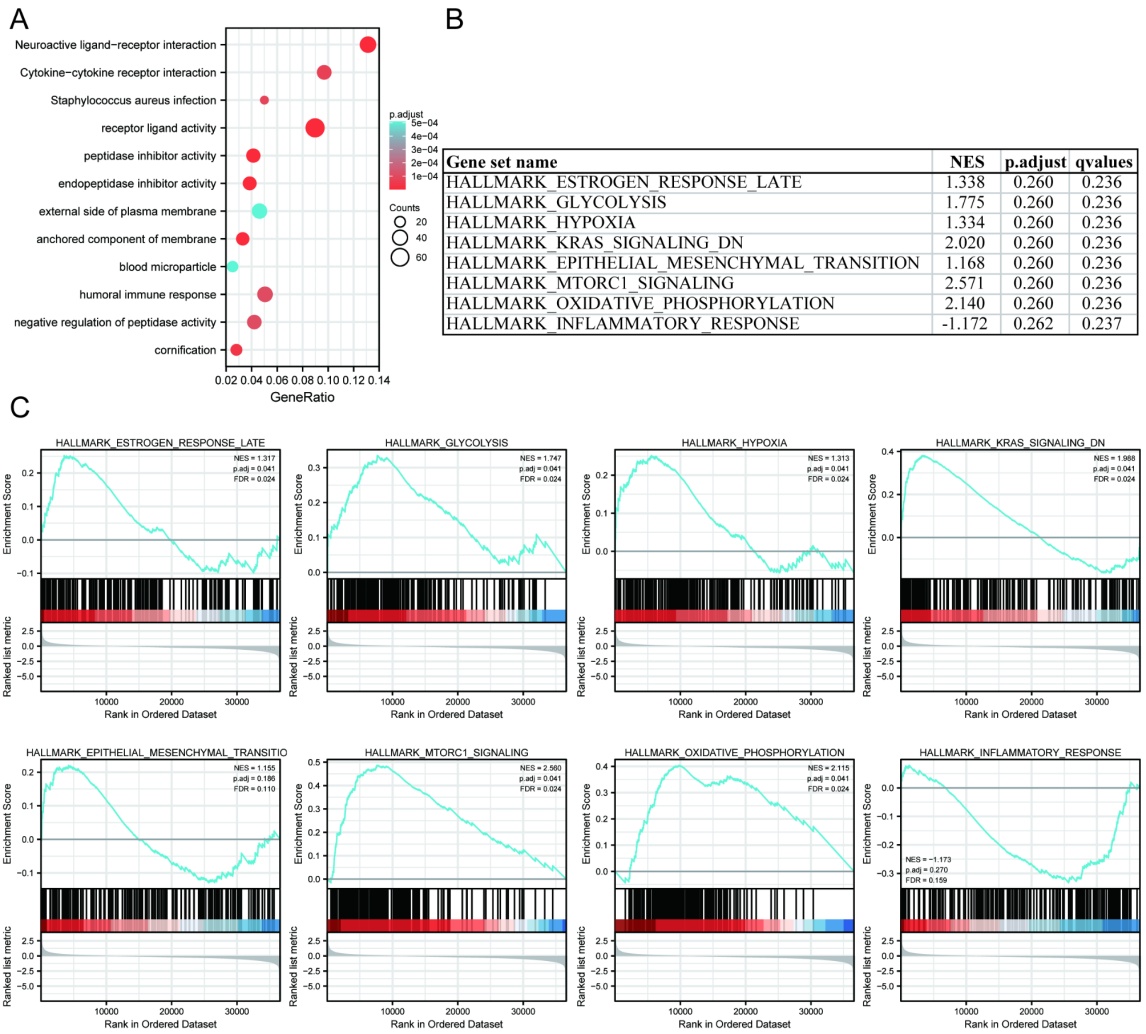
Functional enrichment analysis of PHGDH in endometrial carcinoma. (A) KEGG and GO pathway analysis. (B, C) Enrichment plots of PHGDH-relevant enrichment pathways in h.all.v7.2.symbols.gmt from GSEA

GO, KEGG pathway analysis, and GSEA were used to identify possible cellular mechanisms for the role of PHGDH in endometrial cancer. As shown in Fig. 8A, KEGG enrichment analysis showed that Neuroactive ligand-receptor interaction (hsa04080) was the most relevant pathway to the PHGDH high expression group. At the same time, Cytokine-cytokine receptor interaction (hsa04060) and Staphylococcus aureus infection (hsa05150) were also associated with the role of PHGDH in endometrial cancer. The GO pathways related to the position of PHGDH in endometrial cancer include receptor ligand activity (GO:0048018), external side of plasma membrane (GO:0009897), humoral immune response (GO:0006959), etc.

Meanwhile, the GSEA analysis of estrogen response, glycolysis, hypoxia, K-Ras signaling, epithelial mesenchymal transition, mTOR signaling, etc., were the most abundantly differential pathways in the PHGDH high expression phenotype (Fig. 8B, C).

### PHGDH expression is associated with immune signatures in UCEC

**Fig. 9:**
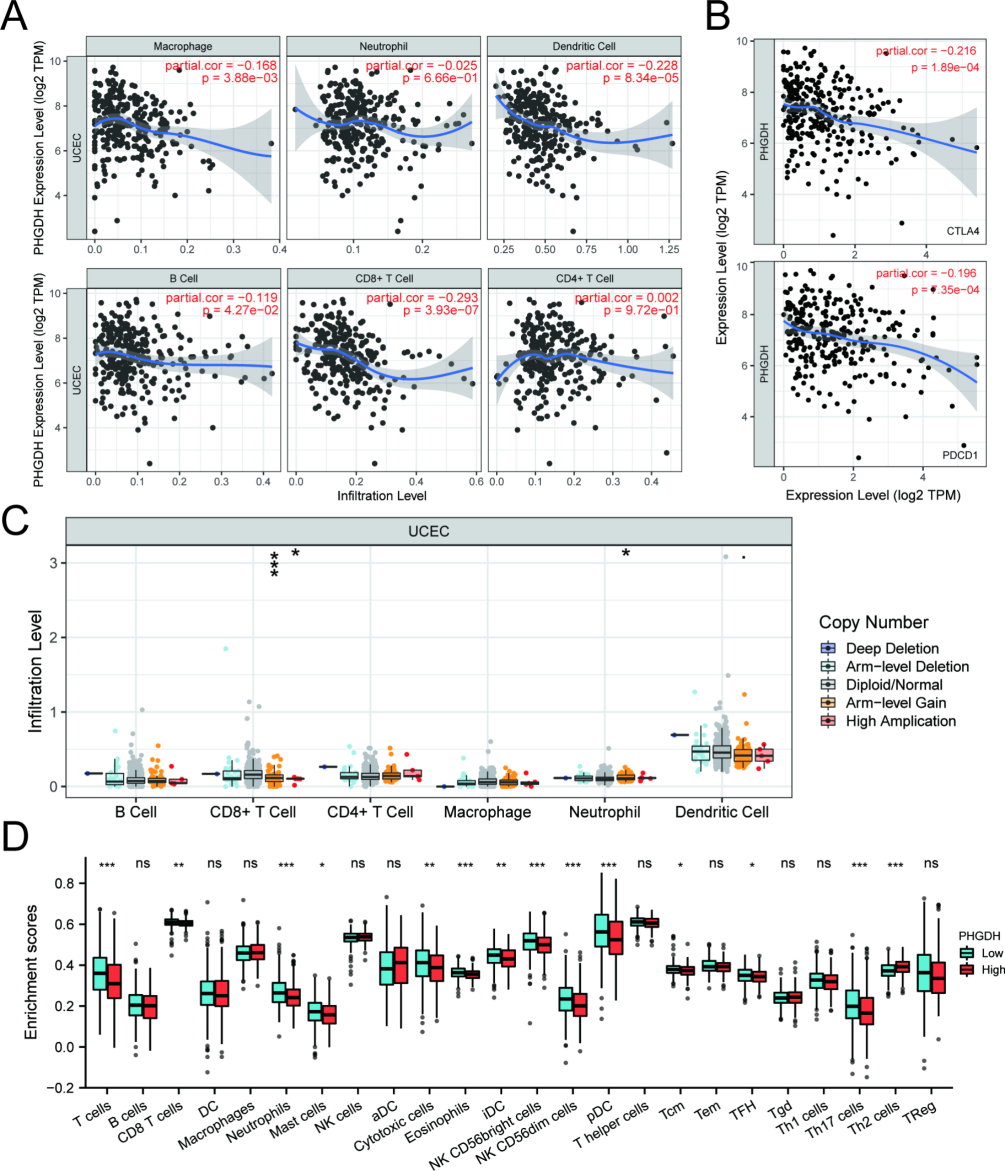
Correlations of PHGDH expression with immune infiltration level in endometrial carcinoma. (A) PHGDH expression was negatively correlated with the levels of macrophage, dendritic cells, B cells and CD8^+^ T cells. (B) PHGDH expression was significantly associated with the immune markers CTLA4 and PDCD1. (C) GLUT1 CNV affects the infiltrating levels of CD8^+^T cells and neutrophils in UCEC. (D) The change ratio of 24 immune cell subtypes in the high and low PHGDH expression groups in UCEC tumor samples

Related studies have shown that tumor immune cell infiltration can be an independent predictor of tumor anterior lymph node status and prognosis [34]. Here, we used TIMER to analyze whether PHGDH expression was associated with the level of immune infiltration in UCEC. As shown in Fig. 9A, PHGDH expression was negatively correlated with the levels of macrophage (P = 0.004), dendritic cells (P < 0.001), B cells (P = 0.043), and CD8^+^ T cells (P < 0.001). These results suggest a crucial role of PHGDH in the immune infiltration of UCEC. In addition, a significant correlation was found between PHGDH CNV and the level of infiltration of CD8^+^ T cells and neutrophils cells (Fig. 9C). PHGDH expression was also significantly associated with the immune markers CTLA4 (P < 0.001) and PDCD1 (PD-1, Programmed cell death protein-1) (P < 0.001) (Fig. 9B).

In addition, we sought to analyze the differential expression of 24 immune cells between different PHGDH expression groups by the CIBERSORT package to determine whether there are differences in the tumor immune microenvironment between high and low PHGDH expression levels in UCEC. The CIBERSORT results showed that the expression of T cells, CD8^+^ T cells, Neutrophils, Mastcells, Cytotoxic cells, Eosinophils, iDC, NK CD56 bright cells, NK CD 56 dim cells, pDC, Tcm, TFH, Th17 cells, and Th2 cells differed more between the high and low PHGDH expression groups. Among them, Th2 cells were increased in the PHGDH high expression group compared to the low expression group, while other cells were decreased in expression (Fig. 9D). These results suggest that PHGDH expression in UCEC is associated with immune cell infiltration in different ways.

### Drug sensitivity analysis of PHGDH

**Fig. 10:**
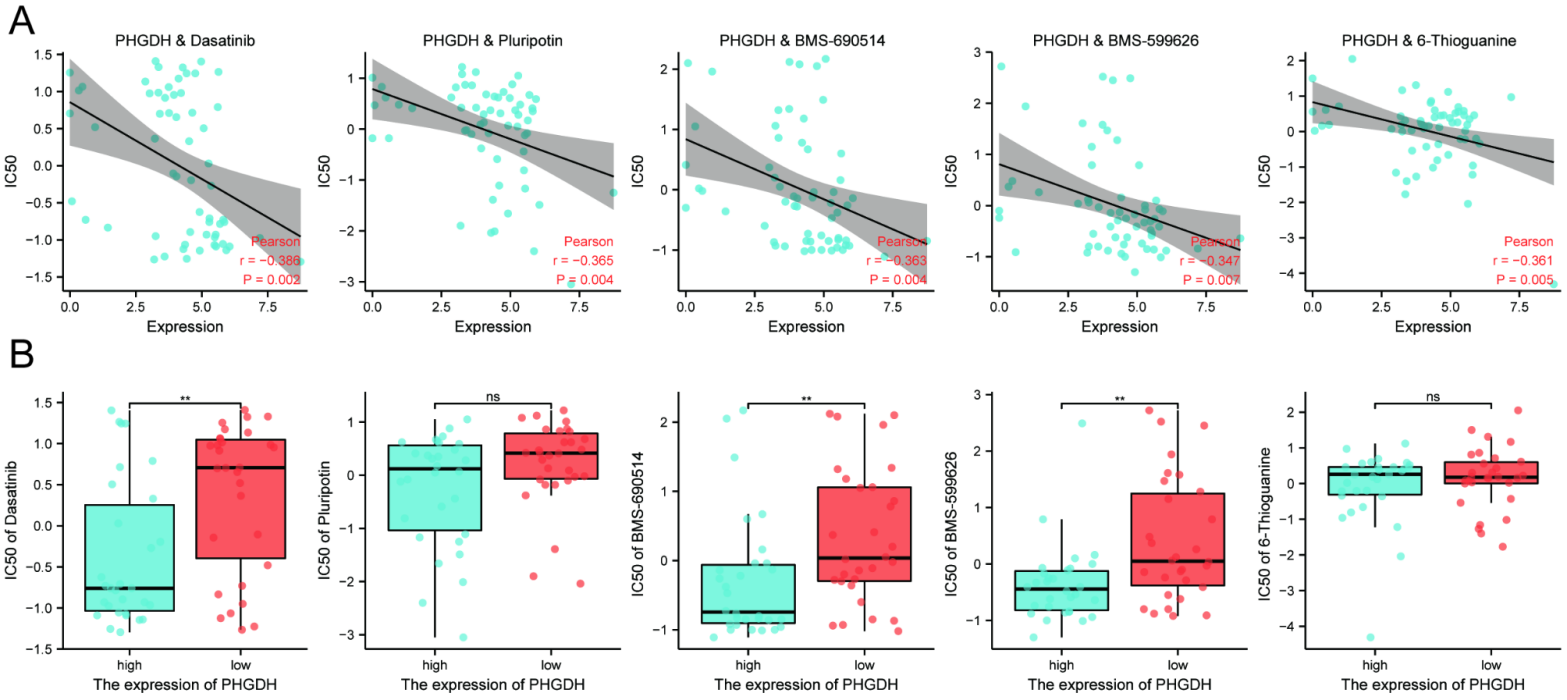
(A) Drug sensitivity analysis of PHGDH from the CellMiner™ database. (B) Differences in drug sensitivity in high versus low PHGDH-expressing groups

Due to the possible drug resistance role of PHGDH in tumors, we further investigated the analysis of potential correlations between drug sensitivity and PHGDH expression using the CellMiner™ database. Our results showed that PHGDH expression was negatively correlated with the sensitivity of Dasatinib (P = 0.002), Pluripotin (P = 0.004), BMS-690,514 (P = 0.004), 6-Thioguanine (P = 0.005) and BMS-599,626 (P = 0.007)(Fig. 10A). Furthermore, there was a significant difference in the expression of Dasatinib (P = 0.007), 6-Thioguanine (P = 0.007), and BMS-599,626 (P = 0.002) in the high versus low PHGDH expression groups (Fig. 10B).

## Discussion

As with most other cancers, the incidence of endometrial cancer and associated mortality rates are on the rise. Due to its complex pathogenesis, it is difficult to develop satisfactory predictive models to predict the overall survival of patients with endometrial cancer. In the past, the prognosis was judged, and treatment options were decided mainly based on the clinical stage of endometrial cancer. In 2013, the molecular typing scheme of endometrial cancer proposed by the Cancer Genome Atlas (TCGA) has provided significant guidance for prognosis and adjuvant therapy of endometrial cancer and promoted our understanding of the biological heterogeneity of endometrial cancer [35]. This has also led to a commitment to a more precise search for new prognostic biomarkers.

Serine is the third most metabolically relevant substance in cancer cells after glucose and glutamine. Phosphoglycerate dehydrogenase (PHGDH) is the first branching enzyme in the serine biosynthetic pathway [10]. Current studies have shown that PHGDH is upregulated in expression in many tumors, and PHGDH inhibitors have been reported to inhibit PHGDH-dependent cancer cell growth and survival, suggesting that it could be a target for cancer therapy [36]. In gynecologic tumors, Zhang et al. found that PHGDH expression was significantly higher in cervical cancer tissues than in normal cervical epithelial cells. In addition, high PHGDH expression in cervical cancer was associated with large tumor size, high FIGO stage, and aggressiveness [37]. Our study showed for the first time that PHGDH expression was significantly upregulated at both mRNA and protein levels in endometrial cancer tissues compared to normal tissues.

The prognostic value of PHGDH in endometrial cancer was presented using the Kaplan-Meier method. The results suggested that the high expression group had poorer OS and DFS than the low PHGDH expression group. Multifactorial COX analysis indicated that high expression of PHGDH could be an independent risk factor for PFI in endometrial cancer patients. The ROC curve subsequently confirmed this. A retrospective analysis analyzed the correlation between glycolipid metabolism and endometrial cancer diagnosis and developed a nomogram prediction model [38]. A study by Zhu et al. also evaluated an OS prediction model incorporating patient clinical characteristics [39]. In this study, a new nomogram prediction model was constructed using age, clinical stage, histologic grade, tumor aggressiveness, histologic type, and PHGDH levels as indicators, using which can improve the accuracy of identifying high-risk patients. Further studies on the relationship between clinical features and PHGDH gene expression showed that high PHGDH expression was associated with clinical stage, pathological type, and histological grade.

In addition, our GSEA results suggest that PHGDH is involved in relevant signaling pathways by mechanisms that may involve estrogen response, glycolysis, hypoxia, K-Ras signaling, epithelial mesenchymal transition, and mTOR signaling. In estrogen receptor-negative breast cancer patients, overexpression of PHGDH leads to poor prognosis, elevated tumor grade, and high expression of proliferation markers and Ki-67 [7, 40]. Disturbed PHGDH expression with altered estrogen receptor-related gene expression has also been seen in endometrial lesions [41]. Since Warburg’s discovery in the 1920s, tumor cells preferentially undergo glycolysis to produce energy and proliferate with greater efficiency under hypoxic conditions, consuming more glucose and producing more lactate (Warburg effect) [42]. We found that PHGDH may be related to energy supply and glycolysis of endometrial cancer cells under hypoxic conditions.

Yesim’s study showed that knockdown of ESRP1 resulted in reduced expression of PHGDH at the mRNA and protein levels. Epithelial splicing regulatory proteins 1 and 2 (ESRP1 and ESRP2) manipulate tumor epithelial-to-mesenchymal transition (EMT). This is also a side effect of the possible association of PHGDH with tumorigenic EMT [43]. Ma et al. demonstrated that glucose restriction induces PHGDH phosphorylation by p38 at Ser371, and in clinical pancreatic cancer specimens, the phosphorylation levels of PHGDH-Ser371 and PHGDH-Ser55 correlated with p38 and AMPK activity, respectively [44]. PHGDH may be associated with tumorigenic EMT through its association with The MAPK signaling pathway interacting with PI3K/AKT/mTOR signaling pathway may jointly promote tumor development [45, 46], suggesting that PHGDH may act through different mechanisms [47]. Although the current study revealed a potential interaction between PHGDH and AMPK signaling pathway, further studies are needed to investigate how PHGDH precisely regulates the development of endometrial cancer.

Naive CD8^+^T cells that differentiate into effector T cells can increase glucose uptake and shift from resting to anabolic metabolism [48]. Glucose-dependent serine biosynthesis mediated by the PHGDH enzyme is essential for CD8^+^T cell expansion in vivo [49, 50]. We used the TIMER database to reveal the relationship between PHGDH expression and immune infiltration levels in UCEC. We found that PHGDH expression significantly correlated with macrophage, dendritic cells, B cells, and CD8^+^T cells. There was also a significant correlation between PHGDH CNV and the level of infiltration of CD8^+^T cells and neutrophils. These results suggest an essential role of PHGDH expression in the regulation of UCEC tumor immunity. We also found that the expression levels of immune cells, such as T cells and CD8^+^T cells, were decreased in the PHGDH high expression group, while the expression levels of Th2 cells were increased. We suggest that PHGDH can affect the immune infiltration of UCEC by regulating CD8^+^T cells. We can speculate that overexpression of PHGDH suppressed the immune response and infiltration of CD8^+^T cells. We suggest that excessive PHGDH in UCEC patients may trigger competition between T cells and tumor cells in the tumor microenvironment, depleting T cells and thus suppressing the antitumor immune response. These findings suggest that PHGDH plays a vital role in the immune infiltrating cells of UCEC. However, controlled and clinical trials are needed to explain the relationship between PHGDH and immune cells such as CD8^+^T cells in vivo.

Drug sensitivity is another important aspect of oncology research. Previous studies have suggested that PHGDH may be associated with tumor resistance. Wei et al. hypothesized that PHGDH is activated in HCC cells in response to sorafenib-induced oxidative stress, and thus PHGDH may be a key driver of sorafenib resistance [51]. Zhang et al. found that inhibition of PHGDH led to doxorubicin-induced oxidative stress and increased doxorubicin sensitivity [52]. In addition, the Knockdown or silencing of PHGDH showed significant antitumor effects in vitro and in vivo, indicating that PHGDH is a promising drug target for tumor therapy [53]. To date, several types of PHGDH inhibitors have been identified as important and emerging options for anticancer therapy.

Our findings suggest that PHGDH can reduce the sensitivity of various targeted drugs. This may partially reveal the mechanism of drug resistance in tumors. Many articles published in recent years have investigated the prognosis-related biomarkers of endometrial cancer and established related predictive models. Geng et al. showed that RNF183 is a prognosis-related molecular marker of endometrial cancer and is associated with its immune infiltration [54]. The study by Chen et al. identified six genes associated with the prognosis of endometrial cancer, established a prognosis-related signature and further analyzed them [55]. Our study was the first to analyze the relationship between the serine metabolism-related gene PHGDH and the development of endometrial cancer and to develop a nomogram model that included clinical features. The immune infiltration characteristics and sensitive drugs associated with it were also analyzed. Our next studies will further focus on the specific targets of PHGDH’s role in endometrial cancer and its mechanisms. Related studies need to be validated by more intensive in vivo and in vitro assays.

## Conclusion

In summary, the analysis showed that PHGDH expression was higher in endometrial cancer tissues than in normal endometrial tissues and correlated with the prognosis of patients. PHGDH is associated with immune response and immune infiltration in endometrial cancer, especially in CD8^+^ T cells. PHGDH has the potential to be a prognostic indicator for patients with endometrial cancer. Recently, PHGDH inhibitors exhibited potent anticancer activity against PHGDH-dependent cancers by assays in cancer cell lines and transplanted tumors [10]. This suggests that PHGDH inhibitors may act as a new targeted agent to inhibit endometrial cancer growth. PHGDH has also contributed to further our understanding of drug resistance in endometrial cancer. Our study provides new insights into the role of PHGDH in endometrial cancer progression. It may provide practical value for future studies of inhibitors targeting PHGDH in the treatment of endometrial cancer.

## Electronic supplementary material

Below is the link to the electronic supplementary material.

Supplementary Material 1

Supplementary Material 2

Supplementary Material 3

## Data Availability

The datasets generated during and analyzed during the current study are available in The Cancer Genome Atlas repository (https://portal.gdc.cancer.gov/). The source codes supporting the conclusions of this article are available on GitHub (https://github.com/zhanghe54321/PHGDH).

## Abbreviations

PHGDH: Phosphoglycerate Dehydrogenase
TCGA: the Cancer Genome Atlas
GSEA: gene set enrichment assay
GO: Gene Ontology Resource
KEGG: Kyoto Encyclopedia of Genes and Genomes
CPTAC: Clinical Proteomic Tumor Analysis Consortium
HPA: The Human Protein Atlas
OS: overall survival
DFS: disease free survival
GEO: Gene Expression Omnibus
EGA: European Genome-phenome Archive
NES: normalized enrichment score
HR: hazard ratios
CI: confidence interval
TPM: Transcripts Per Kilobase of exon model per Million mapped reads
ROC: receiver operating characteristic
AUC: area under the curve
DFI: Disease Free Interval
EMT: epithelial mesenchymal transition
