# Supplementary material for: Comprehensive analysis of PHGDH for predicting prognosis and immunotherapy response in patients with endometrial carcinoma
Source: BMC Med Genomics. 2023 Feb 20;16:29. doi: 10.1186/s12920-023-01463-5 (PMC9942409; doi:10.1186/s12920-023-01463-5)

**Additional file 1** Gene-gene and protein-protein interaction network of PHGDH. (A) The gene network associated with the PHGDH drawn by using GeneMANIA. (B) A network diagram of interactions between proteins encoded PHGDH, drawn by using STRING.


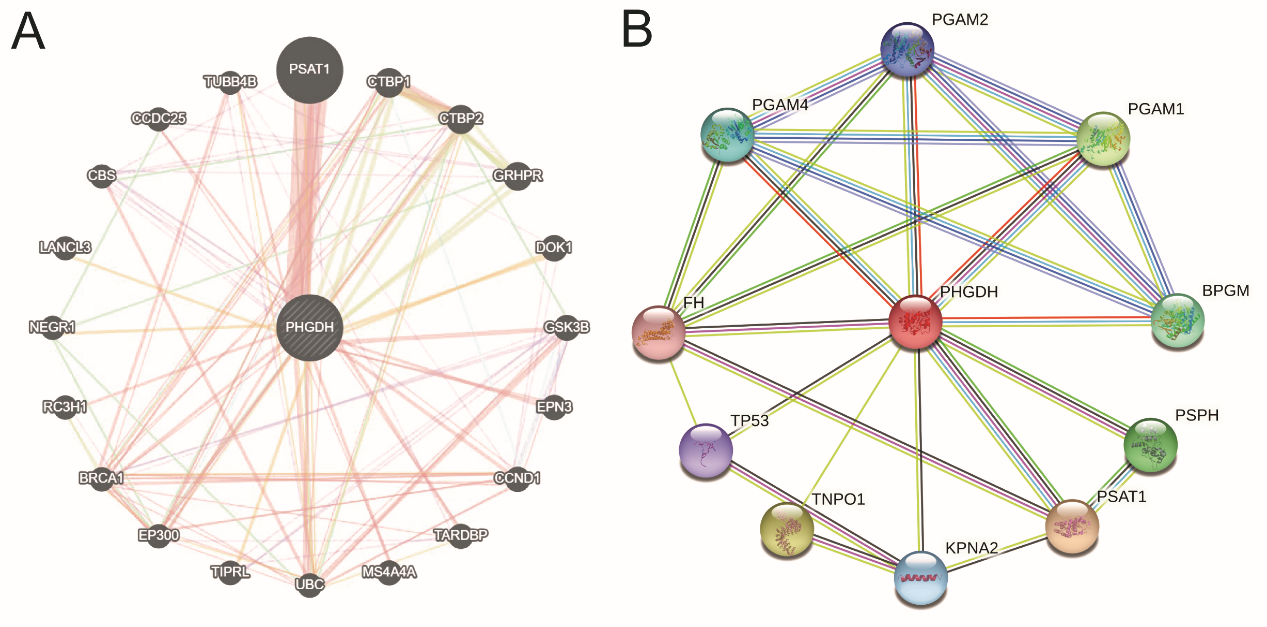

Supplement: Supplementary file 2 — Supplementary Material 2 [file 12920_2023_1463_MOESM2_ESM.docx]
